# Cardiac Cell Tracking with MRI Reporter Genes: Welcoming a New Field

**DOI:** 10.1007/s12410-013-9250-0

**Published:** 2014-01-09

**Authors:** Moriel Vandsburger

**Affiliations:** 1 Department of Physiology, University of Kentucky, Lexington, KY USA; 2 Saha Cardiovascular Research Center, University of Kentucky, 741 South Limestone, BBSRB 355, Lexington, KY 40536 USA

**Keywords:** Magnetic resonance imaging, Molecular imaging, Reporter gene, Cardiac repair, Cell tracking

## Abstract

Research into cell therapy based cardiac repair and regeneration has experienced explosive growth over the last decade, however further progress is hindered by an inability to serially and non-invasively image cell survival and fate decisions following implantation. Recent advances in magnetic resonance imaging (MRI) reporter gene techniques have enabled *in vivo* imaging of cell survival, proliferation, migration, and differentiation, however this has mostly been performed in stationary tissues. A small series of recent studies has examined the possibility of using MRI reporter genes to track the survival of cells injected into the heart following myocardial infarction. In this review, we seek to frame the emerging field of MRI reporter gene based cardiac cell tracking within the larger framework of the needs of cardiac regeneration therapy and the more established field of MRI cell tracking. While initial studies have demonstrated a promising ability to track the viability and proliferation of cells used for cell therapy, the ultimate goal of MR reporter gene imaging in the heart remains the ability to simultaneously correlate cell fate decisions with additional measures of structural and functional recovery.

## Introduction

Research into the use of cell therapy for cardiac regeneration has undergone explosive growth over the prior two decades in both pre-clinical models of infarct healing and in numerous clinical trials. While early studies sought to replace damaged myocardium with implantation of autologous satellite cells (for a fascinating review of early cardiac regeneration research see Yoon et al. [1]), a variety of stem cells with differing origins and levels of pluripotency have been proposed and tested in recent years [2, 3]. Overwhelmingly, most studies have demonstrated a transient improvement in global cardiac function following cell implantation or homing. However, the fraction of cells that survive to later time points is typically below 1 % [4]. The processes underlying the high rates of death amongst injected cells, and potential mechanisms by which to promote increased cell survival and differentiation remain poorly understood. A major barrier to elucidating these processes is the inability to serially examine cell fate decisions following implantation in a non-invasive and *in vivo* manner [5–7].

**Fig. 1:**
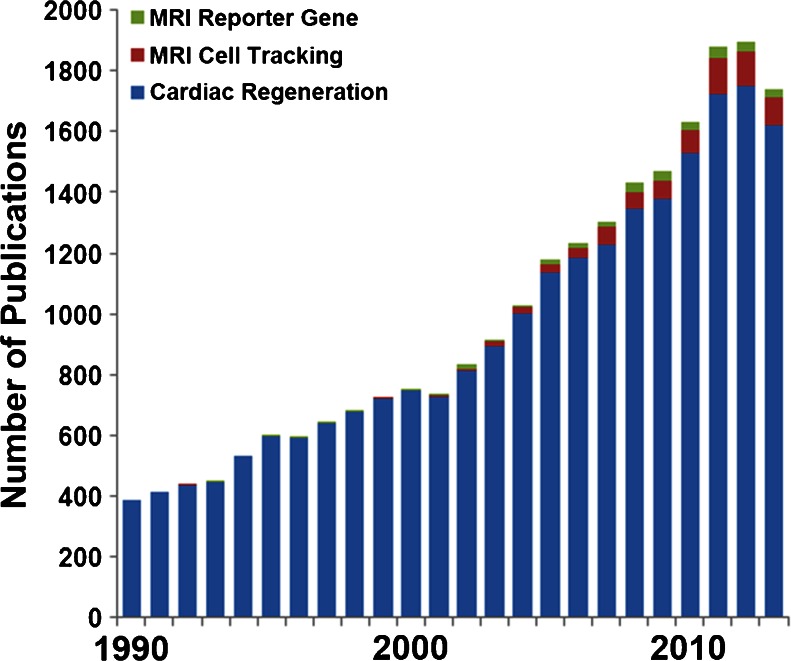
Suever plot of relevant publications. Research into the field of cardiac regeneration with cell therapy has undergone explosive growth since 1990 as evidenced by the large number of publications (blue columns). Overwhelmingly studies have been plagued by extremely low rates of cell survival. An emerging consensus view considers *in vivo* imaging of cell fate decisions as a key tool for improving future therapeutic strategies. MRI cell tracking following cell labeling with exogenous contrast agents (red columns) has emerged over the prior decade as a potential method by which to monitor the survival and viability of injected cells. More recently, the use of MRI reporter genes (green columns) has been explored for *in vivo* imaging of cell survival, proliferation, and differentiation. In the context of MR reporter gene cell tracking in the heart, only four studies have to date been published on imaging the survival of reporter gene expressing cells following intramyocardial injection

Magnetic resonance imaging (MRI) has become a reference standard modality for non-invasive imaging of soft tissue with superior contrast. When applied to imaging of the heart, cardiac MRI (CMR) is emerging as a cutting edge modality capable of enabling multi-scale characterization of the heart from the cellular to the whole organ level [8, 9]. Significant effort has been invested over the past decade in the field of MRI cell tracking, in particular following the labeling of cells with either T1 or T2 shortening contrast agents. However, results of such studies have been mixed, indicating that cell labeling provides inconsistent assessment of cell survival. Recent advances in MR reporter gene technology have enabled *in vivo* imaging of cell survival, migration, and differentiation primarily in stationary organs and tumors (for a full review see ref [10]). Application of MR reporter gene imaging to the heart could transform cardiac regeneration research by enabling direct correlation of injected cell survival with important therapeutic outcomes such as restoration of contractile function and perfusion. Paradoxically, studies of cell based cardiac regeneration therapy now number in the thousands, yet studies utilizing MR reporter gene imaging of cell survival following intramyocardial transplantation number in the single digits (Fig. 1).

In this review we seek to discuss existing methods for MRI cell tracking and the results of early studies of MR reporter gene cell tracking in the heart. Given the small number of studies performed to date, each study is discussed in detail in order to highlight the important contributions made to the nascent field, and further questions that have arisen as a result of such studies. Finally, we will discuss how important findings from MR reporter gene studies in stationary organs and tumors can potentially lead to more dynamic CMR reporter gene imaging.

## Conventional MRI Cell Tracking

MRI cell tracking has historically relied upon labeling of cells with chemical agents that generate contrast via modulation of either T1 (bright contrast) or T2 (dark contrast) relaxation times [11], or through selective excitation and transfer of saturated magnetization (false color contrast) [12]. For example, labeling of fibroblasts with gadolinium-DTPA, a T1-shortening contrast agent, has been used to track the recruitment of fibroblasts to ovarian carcinoma tumors [13], and to track macrophage migration to the forming scar after myocardial infarction in the mouse heart [14]. The recruitment of gadolinium labeled cells can either be visualized as positive contrast on heavily T1-weighted images, or can be measured by quantifying changes in tissue T1-relaxation times between areas with and without labeled cells. A more widely used cell labeling technique has involved the use of a large variety of iron oxide nanoparticles with a range of sizes and relaxivities [15]. Once internalized, iron oxide nanoparticles de-phase the surrounding magnetic field, leading to significantly shortened T2 relaxation times in the vicinity of labeled cells. Labeled cells can then be easily tracked as signal voids on T2* weighted images, or more quantitatively as areas of reduced T2-relaxation on T2-maps. While detection of cells labeled with T1 or T2 shortening contrast agents can be accomplished using conventional pulse sequences, the use of such agents, and in particular T2 shortening agents, can fundamentally limit the ability to image underlying anatomy or to combine cell tracking with additional measures of contractile function and perfusion. In addition concerns of asymmetric distribution of label in dividing cells, eventual dilution of the cell label, altered cell function following labeling, and in the case of iron oxide nanoparticles retention of label in damaged tissue long after the death of labeled cells [16, 17], all remain significant barriers to MRI cell tracking with conventional contrast agents [17].

An alternative cell labeling strategy that has recently emerged is to label cells with a class of contrast agents that selectively generate contrast through a process termed chemical exchange saturation transfer (CEST) [18–20]. CEST agents resonate at unique frequencies offset from water resonance. Following RF irradiation at a given CEST agent’s resonant frequency, the exchange of saturated magnetization with surrounding bulk water leads to a reduction of the on-resonance water signal. In juxtaposition to conventional T1/T2 shortening contrast agents, the contrast generated by CEST agents can be selectively “turned on”. As a result, multiple cell populations that are labeled with distinct CEST agents can be visualized in a multi-spectral manner, as was recently performed by Ferrauto et al. [19]. Since the contrast from such labeling approaches does not fundamentally disturb the underlying anatomical integrity, CEST-MRI could be combined with additional measures of cardiac structure and function. However, concerns surrounding label dilution, division, and retention remain relevant.

MRI reporter genes can uniquely overcome several of the barriers to conventional cell labeling strategies. First, when initiated with proper genetic reprogramming, MR reporter gene expression will remain constant across successive cell divisions. This eliminates concerns of cell division mediated dilution of contrast agents. Second, since transcription of the reporter gene occurs immediately after division, asymmetric division of an exogenous cell label is removed as a limiting factor to cell tracking. Finally, since production of the MR reporter gene product ceases upon cell death, the likelihood of detecting a false positive signal after cell death is significantly reduced. While such characteristics are highly favorable, it is important to note that many of the first generation of MR reporter genes typically demonstrate lower sensitivity when compared to conventional cell labeling strategies.

## MR Reporter Gene Strategies

**Fig. 2:**
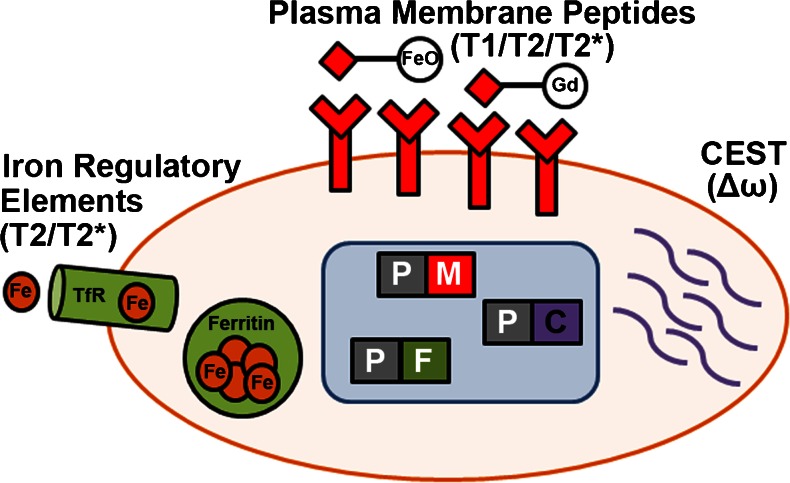
Common MRI reporter gene mechanisms. MRI reporter gene studies have overwhelmingly used three common strategies for *in vivo* detection of cell fate decisions. A large number of studies have examined over-expression or enhancement of iron regulatory elements including ferritin (responsible for iron storage) and/or the transferrin receptor (responsible for shuttling of iron into cells). Other studies have used expression of reporter genes encoding for artificial plasma membrane peptides or antigens with properly targeted iron oxide nanoparticles (T2/T2* contrast) or gadolinium (T1 contrast) contrast agents. Finally, a series of studies has examined expression of a class of engineered artificial reporter peptides that can selectively generate contrast on MR images following excitation with radiofrequency energy applied at reporter specific offset frequencies and chemical exchange of saturated magnetization (CEST) with surrounding bulk water. In theory, such techniques could be combined in a single cell by placing each reporter gene downstream from a specific promoter (P) of interest. (Fe = iron, TfR = transferrin receptor, FeO = iron oxide nanoparticles, Gd = gadolinium chelates, Δω = offset frequency, P = promoter of interest, M = plasma membrane targeted peptide, F = iron regulatory element of interest, C = artificial CEST reporter gene)

A large number of MR reporter gene strategies, each employing a different reporter mechanism, have been proposed and tested over the past decade (for a full review see ref [10]). Overwhelmingly, three unique strategies have emerged and characterize the majority of MR reporter gene research (Fig. 2). In the first strategy, over-expression of iron regulatory elements such as ferritin and/or the transferrin receptor act to increase cellular iron content, thereby shortening T2 relaxation times and enabling detection of reporter gene expressing cells on T2/T2* weighted images. This strategy has been used to examine migration of reporter gene expressing cells in the brain [21, 22], the recruitment of reporter gene expressing cells to tumors [23], the proliferation of cancer cells [24], and differentiation of endothelial cells in the growing mouse embryo [25]. The second broad strategy that has been pursued utilizes the expression of artificial plasma-membrane peptides or antigens as reporters (for a full review see ref [10]), with subsequent targeting using either T1 or T2 modulating contrast agents (Fig. 2). Such a strategy has been used predominantly to image cancer cell proliferation. Finally, the third major strategy has been to mimic the prior strategies with mechanisms that selectively generate CEST contrast through either constitutive expression of CEST reporter genes [26], or through expression of transgenic kinases for targeted CEST contrast agents [27]. These strategies have largely been used to image cancer cell survival and proliferation following implantation in the rodent brain. Of the more than 30 MR reporter gene studies published to date, only four have focused on dynamic cell tracking in the heart [28, 29••, 30••, 31], and are discussed in greater detail below.

## MR Reporter Gene Imaging of Survival and Proliferation Following Cell Engraftment

MR reporter gene imaging is an exciting new field that is undergoing initial application to cardiac imaging. To date, CMR studies of reporter gene expressing cells have focused on monitoring cell viability and proliferation following engraftment into the rodent heart after surgically induced myocardial infarction. Broadly, these studies have sought to identify reporter gene expressing cells through generation of T2/T2* contrast on gradient echo images of the heart. In one series of studies, contrast was generated through over-expression of iron regulatory elements in order to safely increase intracellular iron content [28, 30••, 31]. In a separate study, a reporter gene encoding for a plasma membrane bound antigen was targeted with intravenously delivered, antibody conjugated iron oxide nanoparticles [29••]. These early studies have demonstrated a consistent ability to visually identify viable cell populations, confirmed continued expression of MR reporter genes following proliferation, and have not demonstrated any alterations in cell function as a result of reporter gene expression.

### Over-Expression of Ferritin as an MR Reporter Gene

In an early study by Naumova et al. [30••] murine skeletal myoblast cells (C2C12 cell line) were transduced to over-express mouse ferritin heavy chain in combination with the hemagglutinin acid (HA) tag under the control of the constitutively active cytomegalovirus (CMV) promoter. Prussian blue staining of isolated cells confirmed enhanced uptake and retention of iron in C2C12 cells which over-expressed ferritin. The over-expression of ferritin did not affect cell viability or rates of cell division, however exposure of cells to ferric citrate (FC) significantly reduced the rate of cell division irrespective of ferritin over-expression. Furthermore, over-expression did not change the differentiation patterns of C2C12 cells, as both normal and ferritin over-expressing C2C12 cells differentiated into multi-nucleated myotubes. Initial *in vitro* phantom studies revealed a 25 % reduction of T2 relaxation times in ferritin over-expressing C2C12 cells when compared to normal C2C12 cells. In order to examine the impact of exposure to an iron rich environment, as would be the case *in vivo*, the authors incubated ferritin over-expressing cells in FC supplemented medium for 48 hours prior to phantom imaging. Exposure to FC prior to imaging led to a further 50 % reduction of T2 relaxation times.

**Fig. 3:**
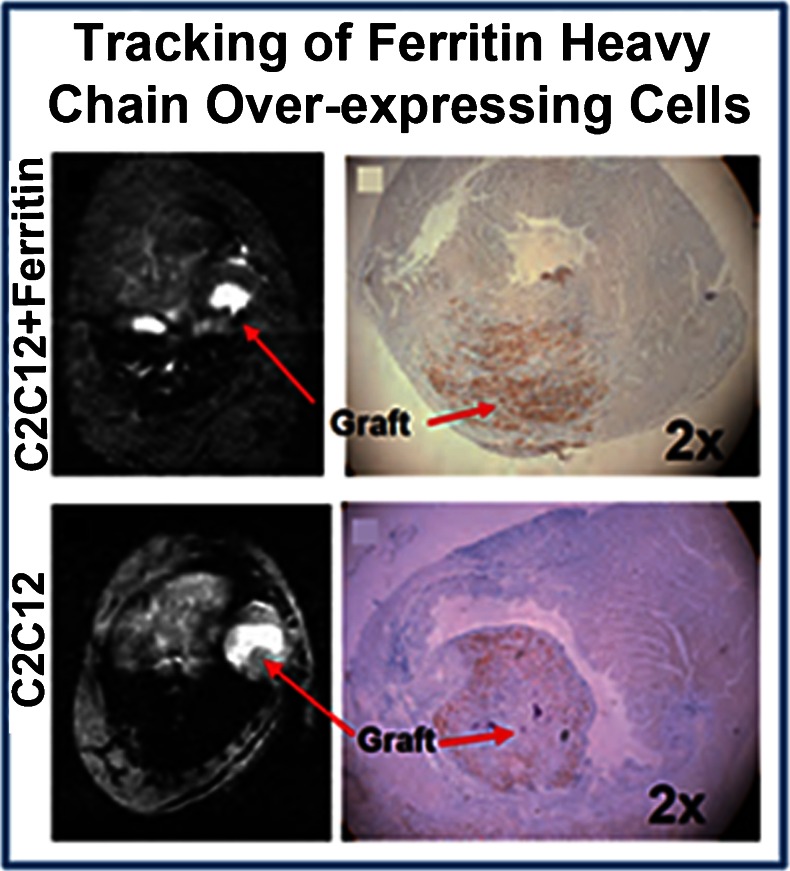
Imaging cell survival and proliferation using ferritin over-expression. Over-expression of ferritin heavy chain in mouse skeletal myoblasts (C2C12 cells) was used as an MR reporter gene strategy to safely increase cellular iron content and shorten T2/T2* relaxation times. Following intramyocardial injection, C2C12 cells that over-express ferritin heavy chain began to accumulate iron concomitant with the process of proliferation. Within three weeks, sufficient iron had accumulated in ferritin over-expressing cells to enable detection as areas of hypo-intense signal using standard cardiac MR gradient echo imaging sequences (red arrow, C2C12 + ferritin). Cells that did not over-express ferritin heavy chain demonstrated similar signal intensity with surrounding myocardial tissue (red arrow, C2C12). Histological assessment of isolated tissue sections confirmed increased iron deposition in grafts of C2C12 cells that over-expressed ferritin. This figure represents work performed by Naumova et al. [30••], but is reprinted with permission from [10]

In order to examine the *in vivo* utility of ferritin over-expression for MR cell tracking, the authors transplanted either C2C12 cells or ferritin over-expressing C2C12 cells into the hearts of C3H mice following surgically induced myocardial infarction (Fig. 3). While initial differences in graft tissue signal intensity were minimal following transplantation, by 3 weeks after implantation ferritin over-expressing C2C12 grafts had accumulated sufficient iron to generate strong signal hypo-intensities on T2* weighted images. In comparison, grafts composed of normal C2C12 cells demonstrated normal signal intensity on identically weighted images. *Ex vivo* staining protocols confirmed that both C2C12 and ferritin over-expressing C2C12 cells had differentiated into skeletal myoblasts replete with sarcomeric proteins. In a subsequent follow up study Naumova and colleagues demonstrated that ferritin over-expressing cells were detected most easily when using standard bright-blood T2* weighted gradient echo imaging, as opposed to black-blood gradient echo or proton density weighted spin echo imaging protocols [31]. Assessment of the size of ferritin over-expressing C2C12 grafts at 4 weeks after transplantation by MRI correlated strongly with graft size as assessed by histology.

In a similar study, over-expression of human ferritin heavy chain was used as a reporter gene for MR tracking of swine cardiac stem cells following implantation into the infarcted rat heart [28]. Campan et al. first used a lenti-virus to induce over-expression of human ferritin heavy chain, and subsequently cultured transgenic cardiac stem cells to form cardiospheres. In agreement with the findings of Naumova et al. [30••, 31], the authors found that over-expression of ferritin heavy chain did not affect cell viability or proliferation. Further, the authors demonstrated that over-expression of human ferritin heavy chain did not change the expression levels of important cell surface markers of multi-potency and cardiac lineage. For *in vivo* rat studies, animals either underwent surgically induced myocardial infarction or sham surgery, and received injections of either phosphate buffered saline (PBS), normal cardiospheres, or ferritin over-expressing cardio-spheres targeted to border zone tissue. The presence of ferritin heavy chain over-expressing cardiospheres was visualized using standard T2*-weighted imaging as early as 1 week after implantation, and remained constant for 4 weeks after implantation in all rats that received ferritin over-expressing cardiospheres regardless of myocardial infarction status. In animals that underwent myocardial infarction surgery, injection of cardiospheres improved LV ejection fraction at four weeks after MI, with no differences observed between ferritin heavy chain over-expressing and wild type cardiospheres. Prussian blue staining of isolated tissue sections confirmed elevated iron deposits in ferritin heavy chain over-expressing cardiospheres 4 weeks following implantation. Importantly, repeated assessment after *in vivo* experiments of viability, proliferation, and key cell surface markers demonstrated no difference between ferritin heavy chain over-expressing and wild type cardiospheres.

These promising early results demonstrate the ability to track the survival and proliferation of cells implanted into the healing infarct using ferritin over-expression as an MRI reporter gene strategy. In both studies, ferritin over-expressing cells appeared as clear regions of signal voids on bright blood T2*-weighted gradient echo images, mimicking the effect of cell labeling with iron oxide nanoparticles. Interestingly, despite relatively similar quantities of injected cells, the signal voids generated by injection of C2C12 cells [30••, 31] were more pronounced than those generated following injection of cardiospheres [28]. These small differences raise the important point that future studies should aspire to create a system by which ferritin over-expression generates consistent T2* contrast across a wide array of candidate cells for cardiac regeneration. Importantly, sufficient accumulation of iron in ferritin over-expressing grafts occurred in both studies, within 3 weeks when using C2C12 cells [30••, 31] and 1 week when using cardiospheres [28]. The period required for sufficient iron accumulation falls well within the likely time between clinical visits for patients of future cell therapy trials. Further, Naumova and colleagues noted that unlike cell labeling strategies, over-expression of ferritin was maintained during the time-course of cell division and replication, leading to a reliable and consistent signal from surviving engrafted tissue [30••, 31]. It is important to note that imaging in both studies was performed on clinical scanners that required specialized small animal inserts. As a result, the spatial resolution of acquired images was lower that what is typically acquired in pre-clinical cell tracking studies, raising the possibility that estimation of graft size and cell proliferation from MR images could have been influenced by partial volume effects. Acquisition of image data at higher spatial resolutions using dedicated small animal MR scanners, or performing similar studies in the hearts of larger animals could mitigate such concerns and further demonstrate the utility of ferritin over-expression as an MRI reporter gene method for cardiac cell tracking.

### Targeting of Plasma Membrane Bound Reporter Antigens

**Fig. 4:**
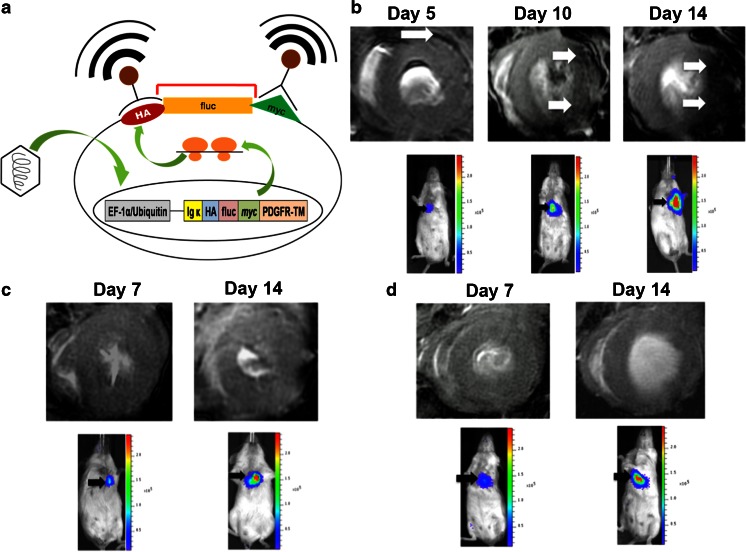
Imaging embryonic stem cell proliferation using over-expression of a plasma membrane antigen with targeted MRI. (**a**) Embryonic stem cells (ESC) were engineered to express hemagglutinin acid (HA) and myc antigens at the plasma membrane. When super-paramagnetic iron oxide nanoparticles were properly targeted to HA and myc antigens, robust T2* contrast could be generated in areas of reporter gene expressing cells. (**b**) HA and myc expressing ESC were implanted into the infarct zone of the mouse heart following myocardial infarction and imaged at multiple time points. T2*-weighted MR imaging of the heart after intravenous administration of the targeted iron oxide nanoparticles demonstrated signal voids in the presence of proliferating and teratoma forming ESC (white arrows). Reporter gene expressing cells were also detected using bioluminescence imaging, which confirmed increased density of reporter gene expressing cells during teratoma formation. (**c**–**d**) Following administration of either false targeted SPIO-HIS-Mab (**c**) or untargeted SPIO nanoparticles (**d**), no significant loss of signal is seen in the myocardium of hearts receiving transplantation of HA and myc expressing ESC. The presence of a clear signal on bioluminescence imaging confirms the presence of the ESC and further confirms the selective targeting of HA and myc antigens. This material was reproduced with permission from John Wiley & Sons, Inc. and Chung et al. [29••]

In contrast to the previously described studies which sought to generate T2/T2* contrast via increased ferritin expression and subsequent accumulation of intercellular iron, an alternative mechanism has been to adapt immuno-histochemical staining techniques for use with MRI. Recently, Chung et al. [29••] engineered embryonic stem cells that express HA and myc antigens on the cell surface (Fig. 4). While neither of these antigens independently function as MR specific reporters, Chung et al. covalently bound super-paramagnetic iron oxide (SPIO) nanoparticles to monocloncal antibodies against HA and myc. Initial *in vitro* studies demonstrated significant loss of signal in embryonic stem cells expressing HA and myc antigens when co-cultured with targeted SPIO particles that was not observed in non-transduced cells. Later, *in vivo* studies demonstrated no difference in signal intensity of transplanted cells on T2*-weighted images prior to administration of antibody bound SPIO particles. Studies performed in animals receiving myc and HA positive embryonic stem cells and HA or myc targeted SPIO particles demonstrated reduced signal intensity in areas of injected cells starting at 5 days after cell injection, which was confirmed by whole body bioluminescence imaging. Repeated studies revealed increased binding of targeted SPIO particles between five and ten days after cell implantation as the embryonic stem cells proliferated and began to form teratoma (Fig. 4). Similar studies performed with either falsely targeted or untargeted SPIO particles demonstrated minimal signal loss on T2*-weighted images. Myocardial tissue regions identified as containing large fractions of HA and myc expressing embryonic stem cells at MRI were confirmed using H&E staining of isolated tissue sections. The use of targeted SPIO particles can overcome concerns of limited sensitivity that have been raised surrounding ferritin based reporter gene methods. In addition, the authors injected the targeted SPIO particles between 8-12 hours prior to imaging. This advantage suggests that upon translation, such agents could be injected outside of the scanner room and patients could return hours later for an MR examination. Importantly, Chung et al. [29••] used a cell line known to induce teratoma formation, and did not characterize whether expression of the reporter gene construct altered cellular gene expression. When applied to additional pluripotent cell types, it will be critical to confirm that expressions of the reporter gene construct does not alter processes of cell viability, proliferation, and differentiation. Further, as the authors acknowledge, expression of ectopic antigens may trigger an immune response that could destroy implanted cells when implanted into immuno-competent hosts.

## Taking the Next Steps

As the field of cardiac MRI reporter gene based cell tracking continues to develop, there are several key objectives that must be part of the field’s evolution. First, the need to genetically alter cells in order to express MR reporter genes remains a barrier to translation beyond pre-clinical models. Although early MR reporter gene studies have demonstrated minimal impact on cell viability, proliferation, function, and differentiation, and newer site specific integration techniques for genetic modification of cells demonstrate superb safety profiles [32], thorough examination of the effects of MR reporter gene expression on cell function and proliferation must be performed in long term studies to exclude potentially negative outcomes. Second, MR reporter gene cell tracking in the heart has only used constitutive expression of reporter genes. As the field of cell therapy seeks to promote differentiation of pluripotent cells into a variety of cells including cardiomyocytes and endothelial cells, it will be important to test the ability of MR reporter genes to enable visualization of cell differentiation along different lineages. Finally, amongst the studies performed to date, all have used MR reporter genes that generate loss of signal on T2* weighted images. Ideally, various MR reporter gene strategies (CEST, iron regulatory elements, and/or plasma membrane antigens with targeted T1 and T2* contrast agents) could be combined to elucidate a more complete picture of molecular processes within cells, or to examine the differentiation of stem cells into a variety of terminal cell types by linking each reporter gene to cell type specific promoters.

## Conclusion

MR reporter gene imaging is an exciting and burgeoning field that can potentially enable a multi-scale understanding of how cell fate decisions impact subsequent disease and regenerative processes. Application to cardiac imaging is currently in very early stages. However, as this field continues to evolve, the potential to elucidate multiple aspects of cell fate decisions within the framework of cardiac regeneration becomes increasingly possible. With further development, MR reporter gene imaging can someday be used to investigate multiple aspects of cell survival, proliferation, and differentiation in order to gain a deeper understanding of mechanisms by which to promote stem cell survival and regeneration of myocardial tissue.
